# Integrating Mental Health Into Surgical Care: A Qualitative Study of a Perioperative Mental Health Intervention

**DOI:** 10.1097/AS9.0000000000000675

**Published:** 2026-05-15

**Authors:** Joanna Abraham, Joanne Wang, Alicia Meng, Katherine J. Holzer, Ana Baumann, Mary Politi, Caitlin Bess, Michael S. Avidan, Eric J. Lenze

**Affiliations:** From the * Department of Anesthesiology, Washington University, St. Louis, MO; † Institute for Informatics, Data Science and Biostatistics (I2DB), Washington University, St. Louis, MO; ‡ Department of Surgery, Washington University, St. Louis, MO; § School of Public Health, Washington University, St. Louis, MO; ∥ Department of Psychiatry, Washington University, St. Louis, MO.

**Keywords:** anxiety, behavioral treatment, coordination, depression, interviews, medication counseling, polypharmacy

## Abstract

**Objectives:**

To explore patient experiences with a perioperative mental health intervention (PMHI), understand its perceived impact on emotional health, and identify factors affecting engagement and feasibility.

**Background:**

Depression and anxiety can worsen surgical outcomes in older adults, highlighting the need for perioperative emotional health support. We evaluated a PMHI across 3 linked randomized controlled trials in cardiac, oncologic, and orthopedic surgery. Here, we report qualitative findings highlighting patients’ experiences with the PMHI across trials.

**Methods:**

The study was conducted across 3 hospitals in a Midwestern healthcare network. 112 semi-structured interviews were conducted with older patients undergoing surgeries (31 cardiac, 39 oncologic, and 42 orthopedic), complemented by 5 periodic reflection focus groups with 9 interventionists (4 social workers/counselors and 5 pharmacist team members). An inductive thematic approach was used for analysis.

**Results:**

Patients and interventionists perceived the PMHI positively, identifying numerous beneficial factors such as compassionate care, strong rapport, and thorough medication education. However, patients noted nonbeneficial factors limiting engagement with the PMHI, including fatigue and competing personal priorities. Patients with oncologic or complex cardiac procedures reported the greatest benefit from the PMHI, compared with slightly lower perceived usefulness among orthopedic and simpler cardiac patients. Informed by these findings, we present a conceptual model illustrating beneficial and nonbeneficial factors influencing patient perceptions of the PMHI.

**Conclusions:**

Patients’ positive perceptions of the PMHI and its impact on alleviating depression and anxiety symptoms underscore the value of integrating psychological and pharmacological support into perioperative care for older adults.

## INTRODUCTION

Older adults account for approximately 40% of inpatient surgeries in the United States, a proportion that will rise as the population ages.^1^ Age-related frailty, multimorbidity, and cognitive decline heighten risks for perioperative complications and poor surgical outcomes.^2–6^ Mental health concerns are also prevalent in older surgical patients, with nearly 52% experiencing symptoms of depression or anxiety.^7–9^ These symptoms are linked to prolonged recovery, delirium, persistent pain, longer hospitalizations, and diminished rehabilitation participation.^10–14^

Older adults undergoing cardiac, oncologic, and orthopedic surgeries are especially vulnerable to poor postoperative outcomes,^15–19^ and additional factors such as polypharmacy and potentially inappropriate medications for geriatric populations can complicate symptom management for depression and anxiety.^20^ Prior studies on pharmacologic and behavioral interventions for perioperative mental health have shown promise in mitigating these symptoms but have mostly targeted younger and middle-aged surgical populations. For example, studies have shown that ketamine administered intraoperatively improved postoperative pain, depression, and related biomarkers in breast cancer patients breast cancer patients between 18 and 65 years of age with mild to moderate depression,^21^ while gabapentin administered preoperatively reduced anxiety and pain catastrophizing in noncardiac patients with moderate to high preoperative anxiety.^22^ Meanwhile, nonpharmacological treatments have primarily focused on psychotherapy and educational interventions,^23–25^ with 1 randomized controlled trial (RCT) identifying that coronary artery bypass graft patients who received a cognitive behavioral therapy intervention experienced reduced postoperative depression and anxiety and improved quality of life compared with usual care.^26^

While these interventions were found to improve perioperative outcomes, there are very few interventions explicitly developed for older adults (aged 60 years or older^27^) undergoing surgeries.^28^

To address this gap, our team co-developed a perioperative mental health intervention (PMHI)^29–32^ tailored for older adults undergoing surgeries. The PMHI is comprised of psychological management (PsychMgmt) and medication optimization (MedOpt), led by licensed social workers/counselors (wellness partners) and a group of pharmacists and pharmacy students (pharmacist team), respectively. Our recent study across 3 linked hybrid type 1 RCTs evaluated both the clinical effectiveness and implementation-potential of the PMHI among cardiac, oncologic, and orthopedic surgical cohorts, in comparison to an enhanced usual care treatment, which consisted of usual care combined with written materials on topics such as mindfulness, surgical recovery, sleep hygiene, and cognitive training. Findings indicated that the PMHI reduced depression and anxiety symptoms and was perceived as acceptable, appropriate, and feasible for implementation in the perioperative setting.

In this report, we present our qualitative findings, offering rich and in-depth perspectives from patients and interventionists regarding their experiences with the PMHI, as well as the contextual factors influencing its fidelity and delivery during perioperative care. These insights are critical for understanding the mechanisms underlying the effectiveness and implementability of the PMHI that can inform future adaptations to support sustainability and scalability within and across other healthcare systems.

## METHODS

### Study Setting

Three linked RCTs were conducted at 1 academic and 2 nonacademic hospitals within a large Midwestern healthcare network between November 1, 2022 and March 31, 2025. The protocol for this study has been published, along with a full description of data collection methods.^32^ The study was approved by the Washington University in St. Louis Institutional Review Board and all participants provided written consent.

### Study Design

The hybrid type 1 RCT evaluation study, followed a sequential explanatory mixed-methods approach which integrated quantitative outcomes with qualitative data to interpret both effectiveness and implementation results. We describe details on how qualitative data were collected throughout each RCT and analyzed to address the aims of this study below. We followed the consolidated criteria for reporting qualitative research.^33^

### Perioperative Mental Health Intervention

Following confirmation of trial eligibility and collection of baseline data, participants were randomized 1:1 to an intervention arm or control arm, which consisted of enhanced usual care (eg, usual care with an additional information packet containing mental health resources for patients to read and pursue if desired).

Patients randomized to the intervention arm of each RCT received the PMHI. The PsychMgmt component included a semi-structured psychotherapy program led by a wellness partner, a Masters-level social worker or counselor trained in psychotherapy and mental health. Wellness partners guided patients through their surgical recovery and changing mental and physical health needs using principles of behavioral activation.^34^ The MedOpt component included a medication review conducted by a trained pharmacist or pharmacy student to identify whether any medication changes would be helpful for the patient, such as adjusting suboptimal dosages or discontinuing use of medications that could be harmful to the brain (Supplemental Digital Content 1, **see** https://links.lww.com/AOSO/A609 for additional details on intervention and control arms).

### Participants: Patients

To participate in the RCTs, patients were required to: (1) be ≥60 years old at the time of surgery; (2) be scheduled for a cardiac, oncologic, or orthopedic surgery at one of the included hospitals; and (3) present with a Patient Health Questionnaire Anxiety and Depression Scale score of ≥10.^35^ Recruitment for RCTs began as early as 60 days prior to scheduled surgery, and patient participation continued from enrollment through approximately 3 months following surgery. Patients who were randomized to and completed the PMHI arm of each RCT were then contacted to complete end-of-study interviews (Supplemental Digital Content 2, see https://links.lww.com/AOSO/A610 for eligibility criteria).

### Interventionists: Study Partners

Study partners included our interventionists: social workers and counselors (ie, wellness partners) and pharmacists and pharmacy students (ie, pharmacist team) who delivered the PMHI to patient participants.

### Data Collection

#### Patient Interviews

Following completion of PMHI sessions, patients were invited to participate in optional end-of-study telephone interviews. These interviews explored experiences with PsychMgmt and MedOpt, perceived effectiveness and satisfaction with the PMHI, its potential impact on symptoms of depression and anxiety, relationships with interventionists, and intervention session format, timing, and delivery. The interview guide was developed and refined during a prior PMHI feasibility study guided by the Consolidated Framework for Implementation Research to draw on domains relevant to intervention delivery and experience (eg, feasibility, acceptability, appropriateness, and perceived impact) and interventionist experiences (Supplemental Digital Content 3, see https://links.lww.com/AOSO/A611 for interview guide).^29,36^ The interview guide was designed to be semi-structured to ensure consistency while allowing flexibility to explore individual experiences in depth. Interviews were conducted by 2 research coordinators (CB and AD) trained by a PhD-level researcher with over 17 years of qualitative methods experience (JA), audio-recorded, and transcribed for analysis. Patients were compensated $25.

#### Interventionist Periodic Reflection Focus Groups

Periodic reflection focus groups were conducted with wellness partners and pharmacist team members during and at the end of the study.^37^ During these sessions, interventionists examined challenges and facilitators to delivering PsychMgmt and MedOpt, surgical cohort differences, perceived patient benefits and barriers to intervention use, and adaptations made to accommodate patient needs and preferences (Supplemental Digital Content 4, see https://links.lww.com/AOSO/A612 for periodic reflection focus group guide). Periodic reflection focus groups were facilitated by a PhD-level researcher with focus group experience (JA, AB, and MP), recorded, and transcribed for analysis.

### Data Analysis

#### Patient Interviews

We followed an inductive (data-driven) thematic analysis approach.^38^ Two researchers (JW and CB) independently reviewed transcripts, openly coded 5 initial interviews, and developed a data-driven codebook refined through iterative discussion until consensus and thematic saturation were reached. Overlapping codes were merged, subthemes were identified, and themes were derived from relationships across transcripts. Examples of subthemes related to PMHI experiences included “perceived value of PMHI” and “implementation characteristics of PMHI delivery.” Coding was independently performed by JW (using NVivo) and JA (manually). Codes, subthemes, and themes were discussed among study team members to achieve 100% consensus.

#### Interventionist Periodic Reflection Focus Groups

Periodic reflection focus group data were analyzed using an inductive thematic analysis bottom-up approach. Examples of subthemes related to interventionist experiences included “patient-interventionist interactions” and “previous patient experiences.” Discrepancies were reviewed and discussed to reach consensus, with biweekly debriefing meetings ensuring analytic rigor and reliability.^39^

## RESULTS

Out of 153 patients allocated to the intervention arm of the RCTs, 142 completed the PMHI, with 11 patients becoming ineligible due to canceled surgeries. 112 of 142 patients subsequently participated in end-of-study interviews (31 cardiac, 39 oncologic, and 42 orthopedic), while 30 declined or were unreachable (Table 1). Five periodic reflection focus groups were conducted: 3 with PsychMgmt team (4 wellness partners) and 2 with MedOpt team (5 pharmacist team members).

**TABLE 1.** Participant Demographics

Variable	Cardiac (n = 31)	Oncologic (n = 39)	Orthopedic (n = 42)
Age	72.1 (5.6)	66.6 (5.5)	68.4 (6.1)
Sex at birth			
Female	15 (48%)	34 (87%)	24 (57%)
Male	16 (52%)	5 (13%)	18 (43%)
Race			
White	28 (91%)	32 (82%)	39 (93%)
Black or African American	2 (6%)	7 (18%)	3 (7%)
American Indian/Alaska Native	1 (3%)	0 (0%)	0 (0%)
Ethnicity			
Non-Hispanic/non-Latino	31 (100%)	39 (100%)	42 (100%)

Mean (SD). n (%).

Findings were organized into 3 categories: experiences with PsychMgmt content and delivery, experiences with MedOpt content and delivery, and perceptions of PMHI value and implementation-potential. Within each category, factors supporting effective PMHI implementation, factors hindering effective PMHI implementation, PMHI adaptations, factors influencing perceived PMHI value, and suggestions for improvement were explored further. Supporting quotes are available in Table 2.

**TABLE 2.** Quotations Supporting Patient Experiences with PsychMgmt Content and Delivery

Subthemes	Factors Supporting Effective PMHI Implementation	Factors Hindering Effective PMHI Implementation	Adaptations
Personalized rationale	Q1*. “I’d say [my wellness partner] was good. She was particularly skilled, I have to say and… I never felt as if… she was, you know, going down a checklist. She would be flexible and nuanced and make changes when I think that was appropriate so that’s a real skill and you don’t see it real often.” (Orthopedic-1367*)	-	-
Values and goals	Q2. *“It’s trying to find your new way of life… That was helpful to me to… kind of set some goals that didn’t have to do with, you know, cleaning my house… [My goals] have to be more, like, personal.” (Oncologic-1069*) Q3. *“I think [PsychMgmt] helped prepare me [for my surgery] beforehand and after to set goals for each step of the healing process… I took it really importantly that I had to be accountable to myself and therefore if I made the goal with [my wellness partner] then I needed to fulfill that unless there was extenuating circumstances.” (Cardiac-1867*)	Q17. *“I just thought that it was rather rigid and I wasn’t sure that those questions were always super appropriate... People’s activities change and the pace at which you do things changes, [and I felt that my wellness partner did not take my age into account when asking questions].” (Orthopedic-1367*)	-
Activity scheduling	Q4. *“I was… having trouble sleeping. [My wellness partner] gave me several ideas on how… to quiet my mind and clear my mind from anything I’d be worrying about. And that did work.” (Cardiac-1304*)	Q18. *“One of my patients, said no, he couldn’t do [activity scheduling and monitoring], because he is absolutely busy… He says that would be too much work on him, so he wouldn’t schedule anything... He says he’s already active, and he doesn’t think that he needs to be strict to scheduling things and… [getting] committed to those things.” (WP2, FG1*) Q19. *“So, you know, you’re pretty beat up after surgery... And you know, you don’t have a lot of energy for anything.” (Oncologic-1304*)	Q23. *“Yeah, I honestly think it’s like more common that it’s not somebody who keeps a structured blog…I think it’s a hard time in [patients’ lives] to be that structured.” (WP3, FG3*)
Activity monitoring	Q5. *“This has been easy. Patients have liked it, and some patients already fill out the form before meeting with [wellness partners]... Revisiting goals in every session has also been helpful.” (WP2, Focus Group (FG)4*) Q6. *“A lot of this is providing a lot of encouragement and celebrating wins. [The patients] know that we are a safe space to talk about things like ‘I took a shower when no one was at home.’ We know how hard these things are, so people appreciate that we would cheerlead them. (WP3, FG4*) Q7. *“I start [my patients] with one activity. And let’s say… someone wants to walk more, and they are really very reluctant about it… So, we start with walking… Let’s say 2 days a week. And then as [you] see that they are picking up the rhythm, you start encouraging them to take it to add on more days.” (WP2, FG2*) Q8. *“I think [my wellness partner] straightened me out a couple times. I was getting my priorities too far out, trying to do more than realistically expectations were and he pulled me back in a couple times if that makes sense.” (Cardiac-1928*)	-	-
Rapport and trust	Q9. *“There’s a definite difference between our ages, but I felt like [my wellness partner] was on the same level as me as far as talking to each other about my problems and I felt like I was talking to a best friend… I felt good after talking to her.” (Cardiac-1306*) Q10. *“Actually, my daughter’s been telling me for years that everybody needs to talk to a counselor and I’ve always been like ‘Yeah, yeah, yeah, yeah.’ I found it a lot more helpful than I anticipated so I thought it was very good… In talking with her, it was exactly what I would expect in talking with a therapist.” (Orthopedic-2035*) Q11. *“Well, you know, I’ve got a pain management psychiatrist and then a regular psychiatrist and then a therapist. You know, so it’s kind of like how many people do I need to talk to me about my feelings and about my emotional self, my mental self. Um, but you know I can tell you I like [my wellness partner] a whole lot better than my psychiatrist.” (Oncologic-1242*)	-	-
Compassionate and collaborative care	Q12. *“Do you know [my wellness partner]? She’s like one of the most wonderful people who walks the face of the earth. She’s charming, she’s professional, she puts you at ease by the way she talks. She makes everything that she tells you seem as if it’s within your grasp and she’s incredibly understanding when life gets busy and you don’t have time to apply yourself to those kinds of difficult changes…She’s amazing.” (Cardiac-1699*) Q13. *“Say something to the effect of, you know, I’d like to… collaborate with you to help decrease your anxiety and depression symptoms. What do you think would be helpful for you? And I’ve been using that with a lot of my patients, actually. And so, they will often come up with… things that aren’t necessarily on the list that we went over, but they’ll come up with other things. So I use that. It’s actually been super helpful.” (WP1, FG2*)	-	Q24. *“The underlying assumptions from traditional [behavioral activation] are not a perfect match (for our patients), so… we’re kind of improvising [and adapting how we taught behavioral activation] a little bit based on what we’ve learned so far about the patient.” (WP3, FG1*)
Cohort-specific feedback	Q14. *“[My experience with the PsychMgmt component] was… helpful. It kind of helps when you’re diagnosed with cancer for the third time. You know, there’s a lot of anxiety you know, you’re not sure how bad it’s gonna be, you’re not sure if you’re gonna have to go back on chemo so you know, it’s kind of good to have someone to talk to, to just kind of to be levelheaded and kind of open-minded about things… I feel like [planning and acting upon behavioral changes was helpful]. It helps you just kind of focus your mind and concentrate and relax.” (Oncologic-1050*) Q15*. “The [PsychMgmt] activities… it does help get your mind off… Well, like right now I’m getting ready to go through radiation. Sometimes if you don’t have essential things to do, you’ll wind up just lying and staying in bed and not getting up and not doing things. And you… make it to the point where you don’t have the desire for anything anymore and that’s not good.” (Oncologic-1345*) Q16. *“One of my participants had an issue where her self-esteem was low and we were talking about... she was living alone, so, it was hard for her to let people come in and, you know, interact with her. So that [caused] the increase in her depression.” (WP2, FG3*)	Q20*. “I was sick for a long time after surgery. I don’t know if everybody’s that way. When I was in surgery I seen—the day after I got out of intensive care—I seen people leaving in three days, I was in there for two weeks after surgery... [It made it hard to engage].” (Cardiac-1928*) Q21. *“People with more complicated healing surgeries might benefit even more than I did because my healing process was very easy.” (Cardiac-2049*) *Q22. “I don’t think I have any depression… I’m simply pretty happy most of the time… [so it wasn’t as needed].’” (Orthopedic-1612*)	-

### Experiences with PsychMgmt Content and Delivery

#### Factors Supporting Effective PMHI Implementation

All patients (n = 112) appreciated that their wellness partners guided them in choosing a personalized plan rather than adhering to a rigid “checklist” of psychotherapeutic strategies [Table 2, Quote 1–2 (Q1–Q2)]. Several (n = 12) reported valuing that their wellness partners recalled personal details and followed up on prior topics. Consistent biweekly sessions also provided patients with the structure and motivation to take concrete, achievable steps to recovery (Table 2, Q3). In addition to scheduling familiar activities, wellness partners introduced mindfulness exercises and stress management strategies, which improved the well-being of many patients (n = 27) (Table 2, Q4).

Wellness partners stated that regularly revisiting goals, documenting progress, and celebrating successes helped patients stay motivated (Table 2, Q5–Q6). Patients agreed, adding that breaking goals into smaller steps allowed them to recognize their progress (Table 2, Q7). When goals were unrealistic or slow-to-reach, wellness partners provided valuable insight into recovery expectations and aided them in cognitive restructuring toward more realistic mental and physical goals (Table 2, Q8).

All patients (n = 112) emphasized the importance of rapport, with 8 patients likening sessions to speaking with a close friend (Table 2, Q9). They appreciated the balance of professionalism and friendliness that encouraged open discussion. Those new to psychotherapy highlighted the comfort that tailored guidance provided (Table 2, Q10).

Across sessions, patients described wellness partners as compassionate, empathetic, and attentive (Table 2, Q12), valuing the emphasis on collaboration and patient-centered prioritization (Table 2, Q13).

Wellness partners and patients observed that oncologic patients benefited the most, as their therapeutic relationship and mindfulness practices promoted positivity and gratitude (Table 2, Q14). The PMHI was uniquely helpful for oncologic patients due to health-related anxiety, emotional vulnerability, and persistent fatigue from chemotherapy, radiation, and treatment recovery (Table 2, Q15–Q16). PsychMgmt appeared to be especially effective in helping these patients manage stress and foster resilience.

#### Factors Hindering Effective PMHI Implementation

Across the behavioral activation steps, patients mostly reported concerns with identifying personal values and goals and scheduling/tracking activities. Some (n = 9) felt the screening and psychoeducation standardized language and procedures were too rigid, repetitive, or not age-appropriate (Table 2, Q17), preferring tailored prompts and guidance.

While many found the sessions motivating, 9 patients reportedly felt that wellness partners limited their sense of independence, preferring to direct their own recovery and rejecting advice on identifying and scheduling helpful activities. Wellness partners similarly noted that some higher-functioning patients took pride in their self-care abilities and resisted suggestions, occasionally leading to unproductive sessions (Table 2, Q18).

Poor health, such as fatigue, pain, or disrupted sleep, were common barriers to completing goals and scheduled activities (Table 2, Q19). Cohort-specific barriers also emerged: cardiac patients cited postoperative complications as limiting behavioral activation, particularly after complex procedures (Table 2, Q20). Wellness partners speculated that this could be due to prolonged sedation and extended recovery time, while patients with simpler procedures (eg, ablation) felt that PMHI was less necessary (Table 2, Q21). For orthopedic patients, mental well-being was closely tied to regaining function, so sessions often overlapped with physical therapy goals, adding little beyond existing care (Table 2, Q22).

#### Adaptations

Requests for flexibility from patients, alongside feedback from weekly meetings and periodic reflection focus groups, enabled iterative adaptations to the treatment manual. Some patients (n = 6) had trouble tracking their activities using the provided, standardized documentation form and therefore relied on alternative methods like smartphone apps (Table 2, Q23). While wellness partners originally followed 4 behavioral activation steps, they also frequently adapted procedures to use a behavioral activation-inspired approach, aligned with each patient’s preferences to create feasible, individualized care plans (Table 2, Q24). Adaptations allowed many patients (n = 26) to more naturally incorporate activity scheduling and monitoring, rather than strictly adhering to weekly homework and check-ins.

### Experiences with MedOpt Content and Delivery

#### Factors Supporting Effective PMHI Implementation

Most patients (n = 85) reported positive relationships with pharmacist team members, stating that rapport was important for establishing trust. Pharmacist team members agreed, believing that humanizing themselves in conversation aided in rapport-building (Table 3, Q1).

**TABLE 3.** Quotations Supporting Patient Experiences with MedOpt Content and Delivery

Subthemes	Factors Supporting Effective PMHI Implementation	Factors Hindering Effective PMHI Implementation	Adaptations
Rapport and trust	Q1. *“Sometimes patients would ask about [me] and sometimes [I] would share things about [myself]. When they knew about [me], it built trust as well.” (P2, FG4*)	Q10. *“I think they were, you know, trying to help but what they were telling me really didn’t make a lot of sense…I think if you want to change someone’s medication, I think you need to talk to [an external] provider before you start doing that.” (Orthopedic-1270*) Q11. *“Uh, well it was pretty evident that [my MedOpt pharmacist] was pretty new at this and very inexperienced... So… [MedOpt pharmacist] needs to get a little bit more confident.” (Orthopedic-1129*)	-
Compassionate and collaborative care	Q2. *“They were always very nice and tried to be helpful and tried to be very empathetic. They didn’t downgrade [sic] you for taking medicine or say to you ‘Why are you taking that?’ in a questioning way or [try] to make it sound like they were more important than you were, or their knowledge was greater than yours.” (Orthopedic-1028*) Q3. *“It was very helpful and [my MedOpt pharmacist] was fantastic about taking time explaining everything about [my medications]… I felt more reassured talking with [my MedOpt pharmacist] because my old primary care provider never talked to me about it.” (Cardiac-1867*) Q4. *“I’m not gonna tell them to stop taking a medicine… No… it’s of course up to them. So, I always tell them… ‘This is my recommendation, and these are the reasons why. Ultimately, it’s up to you as the patient what you weigh as the risk versus the benefit.’” (P5, FG2*)	Q12. *“I’m still on clonazepam and they wanted to take me off of it because I was over a certain age and it would increase my chance of falling. Well… I still work; I still hike in the woods and do all that kind of stuff. And I talked to my psychiatrist and she’s like ‘No, no, no, no we’re not doing that.’ She said if I was in a nursing home and falling down, yeah ok, but just because I’m [age] doesn’t mean I need to be off the meds… It just made no sense.” (Orthopedic-1270*) Q13. *“What really kind of freaked me out was [my MedOpt pharmacist] wanting to talk to my psychiatrist like I’m not adult enough to be in on the conversation, like they have to sort it out for me. I felt it was kind of insulting… It made me feel like an infant. It’s like ‘I’m sorry, I’m not smart enough to understand medication but my psychiatrist and pharmacist are.’” (Oncologic-1453*)	-
Thorough care	Q5. “*I felt like it was more thorough, for lack of a better word. [My MedOpt pharmacist] seemed to really delve into it, and I came away thinking ‘Wow, ok [the pharmacist] really gave me some good solid information….’ So, I was pleased with our discussion.” (Cardiac-1530*) Q6. *“Patients with lower literacy did not realize that some medications were “as needed.” So that sometimes would open conversations about whether (certain) medications were really needed.” (P4, FG4*) Q7. *“The most successful [instances of MedOpt] were when patients were having side effects... [Explaining,] ‘As you have been on these medications for a long time, as you age, the side effects tend to accumulate’ [helped to convince patients to make medication changes].” (P3, FG4*)	-	Q17. *“Benzos were hard to adapt dose... Sleep medication… So many harmful medications. They used to have problems before and then after surgery it was also harder. We are not sleep experts, so it was one of the biggest challenges before and after surgery for all cohorts... We should have embedded a referral to other resources in the protocol but it was not. We… [worked] with wellness partners to reinforce sleeping patterns. It would have been nice to have a sleep expert in the team.” (P3, FG4*)
Interdisciplinary teamwork	Q8. “*They told me what certain ones did, and I ended up stopping the bupropion. They suggested that I try half dose instead of full dose and that didn’t make a difference, so they recommended to call my doctor to stop, and he agreed so we did.” (Cardiac-1306*) Q9. *“They also liked to hear that their physician was on board. It was hard to close the loop with physician. Even if patients were willing, sometimes they were not able to connect with physicians.” (P3, FG4*)	Q14. “*For whatever reason the side effects [that oxycodone] gives me is panic attacks, anxiety… After that initial call I don’t think I heard from [my MedOpt pharmacist]… I didn’t feel like the pharmacist was any benefit whatsoever other than we’re discussing what I’m taking. To my knowledge, [the pharmacist] had no communication with my primary care physician… when I’m having massive problems with the oxycodone, so I don’t really see what the benefit of [MedOpt] was.” (Orthopedic-1792*)	-
Cohort-specific feedback	-	Q15. *“There may have been a shorter window [of opportunity] with [oncologic patients].” (P3, FG4*) *Q16. “A lot of patients wanted to talk with psychiatrists first before changing medications. I think I saw a lot of like, with the psych meds, a lot of patients wanted to talk to their psychiatrist about it… First, I… tried to send a lot of letters to… psychiatrists… But they were never in [the hospital system], so I don’t think we ever really got any responses from any of them either… A lot of the patients I noticed did not necessarily have a great relationship with their provider. And so they wouldn’t offer to call them and we would never really hear back.” (P1, FG4*)	-

Beyond rapport, pharmacist team members prioritized active listening and compassionate care, helping patients feel comfortable and supported (Table 3, Q2). Several patients (n = 36) described their pharmacist team as more thorough than their outpatient providers (Table 3, Q3), increasing receptivity to suggestions. Pharmacist team members also prioritized patient agency and collaborative decision-making, noting that explicitly emphasizing patients’ final say over medication changes increased buy-in and participation (Table 3, Q4).

Most patients (n = 79) described their PMHI sessions as comparable to, or even of superior quality to, their prior or ongoing experiences with primary care clinicians and psychiatrists, noting in particular the additional time spent by the pharmacist team to provide clear education about all medications, including over-the-counter agents, as well as thorough discussions of potential side effects and drug interactions (Table 3, Q5). Pharmacist team members reported that sessions provided a nonjudgmental space to ask questions and revise medication lists and were most successful when patients recognized potential harms of their medications (Table 3, Q6–Q7).

A few patients (n = 5) also praised the interdisciplinary teamwork between MedOpt and outpatient teams, appreciating when recommendations were discussed with outpatient providers to obtain a second opinion and to keep everyone informed. When suggestions to change or discontinue medications were reinforced by outpatient providers, patients were more likely to engage and follow through, encouraging safe medication practice (Table 3, Q8–Q9).

#### Factors Hindering Effective PMHI Implementation

Some patients (n = 7) trusted their long-term outpatient providers more than pharmacist team members, viewing MedOpt suggestions as redundant (Table 3, Q10). Two patients voiced reservations about pharmacy students’ inexperience and perceived lower quality care (Table 3, Q11).

Some (n = 8) also felt the pharmacist team was not sensitive to patient needs (eg, lifestyle, comorbidities) (Table 3, Q12). One described losing agency when a pharmacist team member spoke directly with their psychiatrist about medication changes, excluding them from the conversation (Table 3, Q13). These experiences occasionally led patients to feel that clinician-patient collaboration was discussed more than practiced.

Others (n = 5) reported poor coordination between pharmacist and outpatient teams, noting inconsistent provider communication (Table 3, Q14). Pharmacist team members acknowledged challenges with reaching outpatient providers (Table 3, Q16), delayed responses from in-hospital teams, and gaps in electronic health record updates. They cited poor timing as a barrier to preoperative medication review, as coordinating medication changes and scheduling review sessions before surgery, especially for oncologic patients, was often not feasible (Table 3, Q15).

#### Adaptations

One ongoing concern for both PsychMgmt and MedOpt interventionists was insufficient sleep resources for patients. Therefore, 1 pharmacist team member suggested incorporating a sleep expert into the team (Table 3, Q17).

### Perceptions of PMHI Value and Implementation-Potential

#### Perceived Benefits

Overall, patients found the PMHI valuable for supporting mental health and physical recovery. Most patients (n = 92) said they would participate again, with some who had previously undergone stressful or complex surgeries wishing they had the PMHI during prior procedures (Table 4, Q1). They described emotional support from their interventionists as crucial to maintaining positivity during recovery (Table 4, Q2–Q3). Some (n = 15) cautiously stated that they would only use the PMHI if the nature of their surgery or personal circumstances required it.

**TABLE 4.** Quotations Supporting Patient Perceptions of PMHI Value and Implementation-Potential

Subthemes	Factors Supporting Effective PMHI Implementation	Factors Hindering Effective PMHI Implementation	Adaptations
Previous patient experiences and external factors	Q1. *“I just wish [the PMHI] had been available [during my first hip surgery] where I would’ve really needed it.” (Orthopedic-1733*) Q2. *“I thought [the PMHI] was really very effective, ‘cause I- I mean it’s not like it’s my first surgical procedure, that period of time right before, sometimes you don’t really know what to expect… I think it helped me have a more positive attitude about it… I felt supported.” (Oncologic-1069*) Q3. *“Well, I had previous experience with recovering from heart surgery… I did physical therapy, but I didn’t have any emotional support at that time, so this was drastically different… I do believe that this is very helpful with that kind of thing.” (Cardiac-1699*)	Q8. *“Well, I question the need for the whole program again maybe it’s because I’m experienced with having surgery before, but I don’t know what the whole purpose is.” (Orthopedic-1999*) Q9. *“For some patients who have… gone through surgery already, they find it like a usual thing and when you tell them... Like when you’re introducing... the personalized rationale, they’re like “I’ve gone through this. I know what to do and I know what to expect, so I don’t think I need to do something new, or something more.” So, it becomes a challenge.” (WP2, FG1*) *Q10. “Just myself, you know, and my life circumstance [made it harder to continue with the PMHI practices]. And I live in a household, so I can’t always do exactly what I want to do when I want to do it. But that’s life for most of us, right?” (Orthopedic-1367*)	-
Patient experiences with interventionist team	Q4*. “[It was helpful for patients to have] someone to talk about possible solutions. And if you did not know what to do, you would come back to the weekly meetings to chat and come up with solutions. Sometimes, thinking about engaging with activities can help. Just the conversation can be a motivator.” (WP2, FG4*)	-	-
Implementation characteristics	Q5*. “You’re baring your soul, it’s like being naked… the last thing you want is somebody else hearing your deepest thoughts, unless you have the kind of confidence that I had in [my wellness partner] that she’s not gonna tell anybody.” (Cardiac-1699*) Q6. *“I think you’re kind of lost after the surgery... I don’t think I was prepared for how this surgery would affect me emotionally, so I think the program was very helpful in that respect, plus just talking to [my wellness partner] and having somebody to share my journey with.” (Orthopedic-1344*) Q7. *“A couple times… we bumped into scheduling conflicts and just moved it around… That kind of flexibility was really good, because people have messes that they got to clean up so the flexibility aspect of that really helps.” (Cardiac-1699*)	Q11. *“My cardiac patients have tended to experience adverse events…. Which sometimes interrupts the regularity of the sessions.” (WP2, FG2*)	Q12. *“I think your program should be introduced by the surgeon’s office… It’s just a matter of providing a pamphlet there and you’d be getting a call from these folks. I think it’d be good to add a little bit of continuity between the surgeon and the after care.” (Orthopedic-1733*)

Most found the PMHI feasible (n = 88), did not consider withdrawing (n = 89), and continued some or all PMHI practices or medications postintervention (n = 103). Wellness partners believed that PsychMgmt sessions offered patients an opportunity to start conversations about mental health, improving motivation and mood (Table 4, Q4). In addition to PsychMgmt, patients credited support from their family and collaboration with therapists, psychiatrists, and physical therapists for their success.

Almost all (n = 93) preferred individual sessions, stating that they felt more comfortable disclosing their needs and personal concerns (Table 4, Q5), and favored the convenience of telephone sessions (n = 97). Postoperative sessions were viewed as most beneficial for processing emotions and adjusting to recovery (Table 4, Q6), though some (n = 19) appreciated preoperative sessions for reducing surgery-related anxiety and clarifying recovery expectations. Flexible scheduling was praised as crucial for continued progress (Table 4, Q7).

#### Nonbeneficial Factors Influencing Perceived PMHI Effectiveness and Implementation

A few patients (n = 5) saw limited benefit, noting that prior surgical experience had already informed them of the perioperative process and coping skills (Table 4, Q8-Q9). A small group of 14 patients noted competing obligations or insufficient time to participate fully (Table 4, Q10). Interventionists also noted scheduling challenges, particularly with cardiac and oncologic patients, due to perioperative complications and demanding treatment schedules (Table 4, Q11).

#### Suggestions for Improvement

To further improve patient engagement and clinician-patient trust, some patients (n = 9) and interventionists recommended greater involvement of outpatient and surgical providers, ideally introducing the PMHI preoperatively through their surgeon or outpatient provider (Table 4, Q12). Nearly half of patients (n = 52) also supported offering virtual sessions, noting that video interaction could strengthen clinician-patient connection.

### Conceptual Model

**FIGURE 1.**
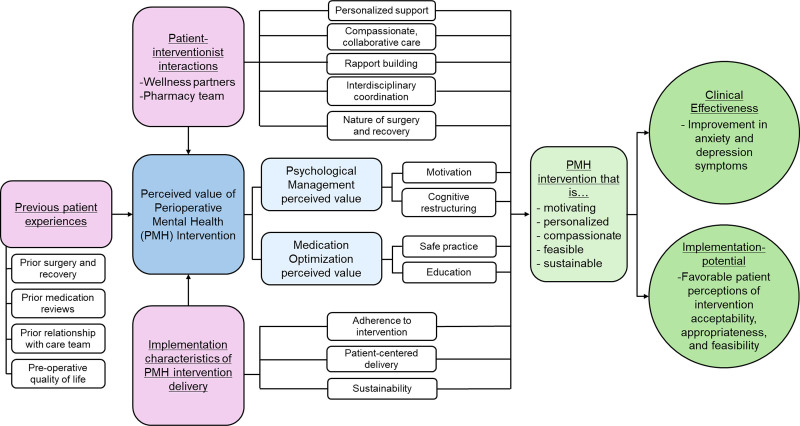
Conceptual model of factors contributing to patients’ perception of PMHI.

We summarize the supporting factors contributing to positive experiences with the PMHI and factors influencing patient perceptions of PMHI value and implementation-potential in the conceptual model (Fig. 1, Supplemental Digital Content 5, see https://links.lww.com/AOSO/A613 for definitions).

This model hypothesizes that patients’ prior experiences, interactions with interventionists, and implementation context shape the perceived value of the PMHI. Patients with positive experiences describe the PMHI as motivating, personalized, compassionate, feasible, and sustainable. The PMHI shows promise for improving symptoms of depression and anxiety and was well-received in terms of acceptability, appropriateness, and feasibility among our target population.

## DISCUSSION

Older adults undergoing surgery often experience symptoms of depression and anxiety that hinder recovery and quality of life.^40^ Given the lack of interventions tailored for older adults undergoing surgeries, we used a community-centered participatory research approach to co-develop a perioperative mental health intervention with patients, interventionists, perioperative clinicians, and treatment developers comprised of a psychological management component supported by behavioral activation and pharmacological management supported by medication optimization.^30^ Our feasibility study with 23 patients indicated positive patient and interventionist perceptions of the intervention, along with preliminary improvements in depression and/or anxiety symptoms.^29^ Informed by these findings, we further tailored our intervention to make it patient-centered, building upon the principles of compassion and care coordination.

Our recent 3 linked RCTs evaluated the effectiveness and implementability of our tailored PMHI among older patients undergoing cardiac, oncology, and orthopedic surgeries.^32^ Initial findings suggested positive patient experiences, with similar fidelity and adherence across cohorts. Notably, the oncology cohort indicated the greatest improvements in depression and anxiety, suggesting intervention benefits may vary by patient context. In this qualitative study, we explored patients’ contextual experiences and perceived impact of the intervention on their emotional health including beneficial and nonbeneficial aspects of the intervention.

We identified 3 key findings which informed a conceptual model to be tested in future work. First, patients viewed the intervention as beneficial. Perceived value was shaped by prior healthcare experiences, patient-interventionist interactions, and implementation context. Second, patients and interventionists noted that the most successful method to promote PMHI engagement and completion was through personalization—emphasizing flexible, individualized delivery. These findings align with reports that targeted, personalized mental health treatments improve outcomes in surgical and older populations.^41–43^ Future studies will explore how to adapt PMHI content to further align with surgery complexity, recovery expectations, and patient needs, particularly for cardiac and orthopedic patients. Third, compassionate care was key to successful patient PMHI engagement. Our patients described interventionists as supportive, active listeners and attributed improved recovery to their strong and trusting relationships with their interventionists, reinforcing evidence that compassionate communication can improve treatment satisfaction and adherence.^44^

These qualitative findings support our initial RCT results on the PMHI’s effectiveness and implementation, providing further explanations for why oncologic patients felt that the intervention was most effective, while orthopedic patients and patients undergoing simpler or highly complex cardiac surgeries did not see significant improvements in their symptoms of depression and anxiety. While most patients generally found the PMHI to be beneficial, we identified surgery-specific contexts to possibly elucidate why oncologic patients showed the largest improvement in symptoms across the study. These patients reported greater isolation and emotional vulnerability due to diagnosis and treatment and found PsychMgmt especially helpful for managing stress, processing emotions, and maintaining hope. These findings are consistent with evidence linking therapeutic engagement to reduced symptoms of depression in oncologic populations, due to the unique risks of cancer recurrence and limited curative options.^45,46^

Cardiac patients undergoing simpler procedures reported that the PMHI would have been more useful for more complex surgeries. This sentiment is also supported by the literature, which suggests that patients undergoing more complex and invasive cardiac procedures experience a higher relative postoperative risk of depression and/or anxiety.^47^ However, our results indicated that high-complexity patients with longer recovery processes also struggled with the PsychMgmt component, as their limited physical ability and additional health issues prevented progress towards goals. Thus, patients with moderately complex cardiac surgeries may benefit most from our PMHI.

Lastly, orthopedic patients prioritized physical recovery, aided by physical therapy and pain management teams, often finding that pain reduction and improved function naturally enhanced mood. Along with speculation from our interventionists and surgeons, our patient findings align with evidence that pain relief and restored mobility are strongly associated with psychological improvement in orthopedic populations and further support our quantitative findings.^48,49^

Overall, PMHI impact varied by surgical complexity and context, with the greatest benefits observed in patients experiencing complicated or prolonged recovery or feelings of uncertainty and isolation. Refining our target population may help to identify individuals most in need of, and most likely to benefit from, the PMHI. Tailoring our PMHI to surgical context and individual needs aligns with the principles of precision mental health,^50^ which emphasizes personalized approaches for vulnerable patients and supports sustainable perioperative mental health integration.^51,52^

Our study has limitations. First, the RCTs were conducted within a single healthcare system, which may have limited patient and clinician perspectives. However, using collaborative planning and community-based participatory research allowed us to co-develop an intervention that was contextually relevant and responsive to our community’s lived experiences.^53,54^ Second, response bias is possible, as patients’ and study partners’ familiarity with the interviewers and focus group moderators may have influenced their responses. Conversely, established rapport may have fostered trust, encouraging patients and study partners to share their thoughts openly. Lastly, approximately 20% of patients did not complete follow-up interviews, raising potential nonresponse bias, though thematic consistency across transcripts reduced the likelihood that perspectives of dissatisfied patients were underrepresented.

In summary, our findings underscore the importance of compassionate, individualized perioperative mental healthcare delivery, while highlighting the need for robust communication strategies to maximize the flexibility and patient-centered nature of our PMHI. Our intervention was perceived as feasible and easy to follow for older adults undergoing surgeries through accessible and personalized delivery across the perioperative care continuum—elements identified as critical to intervention engagement, scalability, and sustainability across populations, and as key drivers of future refinement to enhance effectiveness.

## ACKNOWLEDGMENTS

We acknowledge and thank the study participants for their time and valuable contributions, as well as Acaica Dunkley for her assistance with data collection. We also wish to acknowledge all team members from the Center for Perioperative Mental Health RCT study team for their contributions throughout the study, including Ryan P. Calfee, Simon Haroutounian, Benjamin D. Kozower, Theresa A. Cordner, Emily M. Lenard, Kenneth E. Freedland, Bethany R. Tellor Pennington, Rachel C. Wolfe, J. Philip Miller, Yi Zhang, Michael D. Yingling, Thomas Kannampallil, Julia A. Schweiger, and Sherry L. McKinnon.

## AUTHOR CONTRIBUTIONS

J.A., E.J.L., and M.S.A. conceived the study. C.B. helped with interview data collection, and J.A., A.B. and M.P. facilitated periodic reflection focus groups. J.A. and J.W. conducted the data analysis. J.A., J.W., and A.M. drafted the manuscript. All authors revised the manuscript and approved the final version for submission.

## Supplementary Material

## References

[1] Etzioni DA Liu JH Maggard MA. The aging population and its impact on the surgery workforce. Ann Surg. 2003;238:170–177. 12894008 10.1097/01.SLA.0000081085.98792.3d PMC1422682

[2] Devalapalli AP Kashiwagi DT. Perioperative care of geriatric patients. Hosp Pract (1995). 2020;48:26–36. 31976774 10.1080/21548331.2020.1719713

[3] Ablett AD McCarthy K Carter B. Cognitive impairment is associated with mortality in older adults in the emergency surgical setting: findings from the older persons surgical outcomes collaboration (OPSOC): a prospective cohort study. Surgery. 2019;165:978–984. 30454842 10.1016/j.surg.2018.10.013

[4] Makary MA Segev DL Pronovost PJ. Frailty as a predictor of surgical outcomes in older patients. J Am Coll Surg. 2010;210:901–908. 20510798 10.1016/j.jamcollsurg.2010.01.028

[5] Robinson TN Wu DS Pointer LF. Preoperative cognitive dysfunction is related to adverse postoperative outcomes in the elderly. J Am Coll Surg. 2012;215:12–7; discussion 17. 22626912 10.1016/j.jamcollsurg.2012.02.007 PMC3383613

[6] Rosen CB Wirtalla C Keele LJ. Multimorbidity confers greater risk for older patients in emergency general surgery than the presence of multiple comorbidities: a retrospective observational study. Med Care. 2022;60:616–622. 35640050 10.1097/MLR.0000000000001733 PMC9262850

[7] Alhamdah Y. Examining Depression in Older Surgical Patients: An Observational Cohort Study. University of Toronto (Canada); 2024.

[8] Cenzer I Inouye SK Raue PJ. Trajectories of postoperative depressive symptoms in older patients undergoing major surgery. JAMA Netw Open. 2024;7:e2354154–e2354154. 38294817 10.1001/jamanetworkopen.2023.54154 PMC10831558

[9] Srifuengfung M Abraham J Avidan MS. Perioperative anxiety and depression in older adults: epidemiology and treatment. Am J Geriatr Psychiatry. 2023;31:996–1008. 37482501 10.1016/j.jagp.2023.07.002 PMC10592367

[10] Ghoneim MM O’Hara MW. Depression and postoperative complications: an overview. BMC Surg. 2016;16:1–10. 26830195 10.1186/s12893-016-0120-y PMC4736276

[11] Lenze EJ Munin MC Dew MA. Adverse effects of depression and cognitive impairment on rehabilitation participation and recovery from hip fracture. Int J Geriatr Psychiatry. 2004;19:472–478. 15156549 10.1002/gps.1116

[12] Tang VL Cenzer I McCulloch CE. Preoperative depressive symptoms associated with poor functional recovery after surgery. J Am Geriatr Soc. 2020;68:2814–2821. 32898280 10.1111/jgs.16781 PMC7744402

[13] Yang K-L Detroyer E Van Grootven B. Association between preoperative anxiety and postoperative delirium in older patients: a systematic review and meta-analysis. BMC Geriatr. 2023;23:198. 36997928 10.1186/s12877-023-03923-0 PMC10064748

[14] Orri M Boleslawski E Regimbeau JM. Influence of depression on recovery after major noncardiac surgery: a prospective cohort study. Ann Surg. 2015;262:882–9; discussion 889. 26583680 10.1097/SLA.0000000000001448

[15] Van Cleave JH Egleston BL McCorkle R. Factors affecting recovery of functional status in older adults after cancer surgery. J Am Geriatr Soc. 2011;59:34–43. 21226675 10.1111/j.1532-5415.2010.03210.x PMC3176326

[16] Ghoshal A Bhanvadia S Singh S. Factors associated with persistent postsurgical pain after total knee or hip joint replacement: a systematic review and meta-analysis. Pain Rep. 2023;8:e1052. 36699992 10.1097/PR9.0000000000001052 PMC9833456

[17] Javeed S Benedict B Yakdan S. Implications of preoperative depression for lumbar spine surgery outcomes: a systematic review and meta-analysis. JAMA Netw Open. 2024;7:e2348565–e2348565. 38277149 10.1001/jamanetworkopen.2023.48565 PMC10818221

[18] Tully PJ. Psychological depression and cardiac surgery: a comprehensive review. J Extra Corpor Technol. 2012;44:224–232. 23441564 PMC4557565

[19] Wada S Inoguchi H Sadahiro R. Preoperative anxiety as a predictor of delirium in cancer patients: a prospective observational cohort study. World J Surg. 2019;43:134–142. 30128769 10.1007/s00268-018-4761-0

[20] Kawabata S Michikawa T Nagai S. Possible negative impact of polypharmacy on surgical outcomes in older patients with lumbar spinal stenosis. Geriatr Gerontol Int. 2025;25:31–37. 39586669 10.1111/ggi.15026 PMC11711071

[21] Liu P Li P Li Q. Effect of pretreatment of S-ketamine on postoperative depression for breast cancer patients. J Invest Surg. 2021;34:883–888. 31948296 10.1080/08941939.2019.1710626

[22] Clarke H Kirkham KR Orser BA. Gabapentin reduces preoperative anxiety and pain catastrophizing in highly anxious patients prior to major surgery: a blinded randomized placebo-controlled trial. Can J Anaesth. 2013;60:432–443. 23377862 10.1007/s12630-013-9890-1

[23] Hall AE Nguyen NH Cascavita CT. The impact of psychological prehabilitation on surgical outcomes: a meta-analysis and meta-regression. Ann Surg. 2025;281:928–941. 39969855 10.1097/SLA.0000000000006677

[24] Zhang F Wang L-Y Chen Z-L. Cognitive behavioral therapy achieves better benefits in relieving postoperative pain and improving joint function: a systematic review and meta-analysis of randomized controlled trials. J Orthop Sci. 2024;29:681–689. 36775785 10.1016/j.jos.2023.01.007

[25] Guo P. Preoperative education interventions to reduce anxiety and improve recovery among cardiac surgery patients: a review of randomised controlled trials. J Clin Nurs. 2015;24:34–46. 24894181 10.1111/jocn.12618

[26] Dao TK Youssef NA Armsworth M. Randomized controlled trial of brief cognitive behavioral intervention for depression and anxiety symptoms preoperatively in patients undergoing coronary artery bypass graft surgery. J Thorac Cardiovasc Surg. 2011;142:e109–e115. 21621227 10.1016/j.jtcvs.2011.02.046

[27] United Nations. World Population Ageing 2013. Department of Economic and Social Affairs, Population Division: 2013.

[28] Holzer KJ Avidan MS Lenze EJ. Perioperative mental health in older adults: new research on epidemiology and outcomes. Am J Geriatr Psychiatry. 2021;29:1222–1224. 33653599 10.1016/j.jagp.2021.02.036

[29] Abraham J Holzer KJ Lenard EM. A perioperative mental health intervention for depressed and anxious older surgical patients: results from a feasibility study. Am J Geriatr Psychiatry. 2024;32:205–219. 37798223 10.1016/j.jagp.2023.09.003 PMC10852892

[30] Abraham J Meng A Baumann A. A multi-and mixed-method adaptation study of a patient-centered perioperative mental health intervention bundle. BMC Health Serv Res. 2023;23:1175. 37891574 10.1186/s12913-023-10186-3 PMC10612159

[31] Abraham J Meng A Siraco S. A qualitative study of perioperative depression and anxiety in older adults. Am J Geriatr Psychiatry. 2020;28:1107–1118. 32234274 10.1016/j.jagp.2020.02.010

[32] Holzer KJ Bartosiak KA Calfee RP. Perioperative mental health intervention for depression and anxiety symptoms in older adults study protocol: design and methods for three linked randomised controlled trials. BMJ Open. 2024;14:e082656. 10.1136/bmjopen-2023-082656 PMC11146368 38569683

[33] Tong A Sainsbury P Craig J. Consolidated criteria for reporting qualitative research (COREQ): a 32-item checklist for interviews and focus groups. Int J Qual Health Care. 2007;19:349–357. 17872937 10.1093/intqhc/mzm042

[34] Hopko DR Lejuez C Ruggiero KJ. Contemporary behavioral activation treatments for depression: procedures, principles, and progress. Clin Psychol Rev. 2003;23:699–717. 12971906 10.1016/s0272-7358(03)00070-9

[35] Kroenke K Wu J Yu Z. Patient health questionnaire anxiety and depression scale: initial validation in three clinical trials. Psychosom Med. 2016;78:716–727. 27187854 10.1097/PSY.0000000000000322 PMC4927366

[36] Damschroder LJ Aron DC Keith RE. Fostering implementation of health services research findings into practice: a consolidated framework for advancing implementation science. Implement Sci. 2009;4:50. 19664226 10.1186/1748-5908-4-50 PMC2736161

[37] Finley EP Huynh AK Farmer MM. Periodic reflections: a method of guided discussions for documenting implementation phenomena. BMC Med Res Methodol. 2018;18:153. 30482159 10.1186/s12874-018-0610-y PMC6258449

[38] Braun V Clarke V. Using thematic analysis in psychology. Qual Res Psychol. 2006;3:77–101.

[39] Spall S. Peer debriefing in qualitative research: emerging operational models. Qual Inq. 1998;4:280–292.

[40] Chen A An E Yan E. Prevalence of preoperative depression and adverse outcomes in older patients undergoing elective surgery: a systematic review and meta-analysis. J Clin Anesth. 2024;97:111532. 38936304 10.1016/j.jclinane.2024.111532

[41] Raue PJ Sirey JA. Designing personalized treatment engagement interventions for depressed older adults. Psychiatr Clin North Am. 2011;34:489–500, x. 21536170 10.1016/j.psc.2011.02.011 PMC3087152

[42] von Lützow, U Neuendorf NL Scherr S. Effectiveness of just-in-time adaptive interventions for improving mental health and psychological well-being: a systematic review and meta-analysis. BMJ Mental Health. 2025;28:e301641. 10.1136/bmjment-2025-301641 PMC12481328 41027677

[43] Kondylakis H Giglioli IAC Katehakis D. Stress reduction in perioperative care: feasibility randomized controlled trial. J Med Internet Res. 2025;27:e54049. 39773866 10.2196/54049 PMC11751654

[44] Epstein RM Street RL. The values and value of patient-centered care. Ann Fam Med. 2011;9:100–103. 21403134 10.1370/afm.1239 PMC3056855

[45] Mitchell AJ Chan M Bhatti H. Prevalence of depression, anxiety, and adjustment disorder in oncological, haematological, and palliative-care settings: a meta-analysis of 94 interview-based studies. Lancet Oncol. 2011;12:160–174. 21251875 10.1016/S1470-2045(11)70002-X

[46] Ryba MM Lejuez CW Hopko DR. Behavioral activation for depressed breast cancer patients: the impact of therapeutic compliance and quantity of activities completed on symptom reduction. J Consult Clin Psychol. 2014;82:325–335. 24364801 10.1037/a0035363

[47] Wegermann ZK Mack MJ Arnold SV. Anxiety and depression following aortic valve replacement. J Am Heart Assoc. 2022;11:e024377. 35470691 10.1161/JAHA.121.024377 PMC9238623

[48] Caracciolo B Giaquinto S. Self-perceived distress and self-perceived functional recovery after recent total hip and knee arthroplasty. Arch Gerontol Geriatr. 2005;41:177–181. 16085069 10.1016/j.archger.2005.01.006

[49] Tarakji BA Wynkoop AT Srivastava AK. Improvement in depression and physical health following total joint arthroplasty. J Arthroplasty. 2018;33:2423–2427. 29681494 10.1016/j.arth.2018.03.051

[50] Bickman L Lyon AR Wolpert M. Achieving precision mental health through effective assessment, monitoring, and feedback processes: introduction to the special issue. Adm Policy Ment Health. 2016;43:271–276. 26887937 10.1007/s10488-016-0718-5 PMC4832000

[51] Langford DJ Sideris A Poeran J. Prioritising mental health in the perioperative period: understanding postoperative patterns in anxiety and depression through ecological momentary assessment. Br J Anaesth. 2025;134:19–22. 39756852 10.1016/j.bja.2024.10.010

[52] Wang H Farb N Saab B. Scalable precision psychiatry with an objective measure of psychological stress: prospective real-world study. J Med Internet Res. 2025;27:e56086. 40622759 10.2196/56086 PMC12280831

[53] Cabassa LJ Gomes AP Meyreles Q. Using the collaborative intervention planning framework to adapt a health-care manager intervention to a new population and provider group to improve the health of people with serious mental illness. Implement Sci. 2014;9:178. 25433494 10.1186/s13012-014-0178-9 PMC4255430

[54] Jones DE Lindquist-Grantz R DeJonckheere M. A review of mixed methods community-based participatory research applications in mental health. JSBHS. 2020;14:254–288.

