# Supplementary material for: Integrating Mental Health Into Surgical Care: A Qualitative Study of a Perioperative Mental Health Intervention
Source: Ann Surg Open. 2026 May 15;7(2):e675. doi: 10.1097/AS9.0000000000000675 (PMC13290208; doi:10.1097/AS9.0000000000000675)
Supplement: Supplementary file 1 [file as9-7-e675-s001.pdf]

Supplement 1. Details on Intervention and Control Arms of RCT.

Intervention

Participants randomized to the intervention arm of each RCT received the perioperative mental health (PMH) intervention, which consisted of two components. First, the psychological management (PsychMgmt) component was a semi-structured psychotherapy program, led by a Masters-level social worker or counselor trained on psychotherapy and mental health – also called a Wellness Partner. Wellness Partners guided them through their surgical recovery and changing mental and physical health needs. The PsychMgmt component guided participants through the principles of behavioral activation [1], a strategy to mitigate symptoms of anxiety and depression through engagement in meaningful activities. Behavioral activation was taught in four steps: 1) developing a personalized rationale to practice introspection and needs assessment; 2) assessing personal values and goals; 3) scheduling activities that align with patient goals; and 4) monitoring activities. Throughout the process, Wellness Partners utilized principles of compassionate care and care coordination [2], allowing participants to tailor behavioral activation and choose goals and activities that they found personally rewarding.

Second, the medication optimization (MedOpt) component provided an optional opportunity for a pharmacist/pharmacy student to conduct a medication review and identify whether any medication changes would be helpful for the participant (e.g., adjusting suboptimal dosages, discontinuing use of medications that could be harmful to the brain). First, the participant's home medications were reviewed and verified (e.g., doses and frequencies). If the participant was taking a targeted, potentially inappropriate central nervous system active medication from the pharmacist's list of "Targeted Harmful Medications for Optimization," the pharmacist would inform the participant about why their medication was targeted. They would then develop a personalized deprescribing plan according to the participant's interest in a medication adjustment (or discontinuation), along with their likely need for the change. Additionally, the pharmacist would recommend dose optimization for any subtherapeutic antidepressants from the list of "Antidepressant Medications of Interest." The pharmacist and participant would collaboratively discuss recommended changes alongside outpatient prescribing providers, if necessary. If the participant and outpatient provider agreed, the medication change and new medication plan were implemented, and in following sessions (up to 3 months postoperatively), the participant's response to the medication changes were monitored. Additionally, while the participant was in the hospital, the MedOpt pharmacist coordinated with inpatient care team members to ensure that the participant was taking the correct medications, and no new inappropriate medications were introduced.

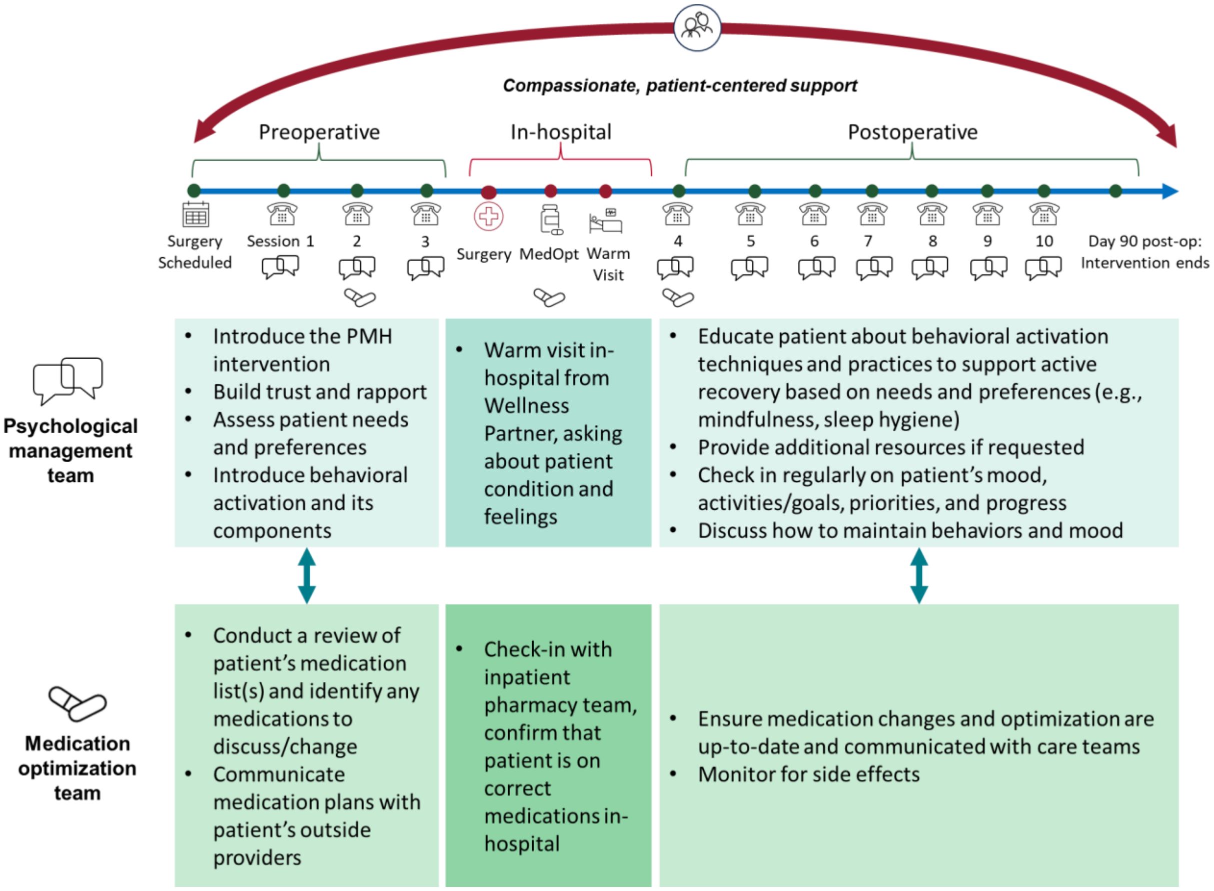

Figure 1. PMHI Timeline and Components.

Both components also emphasized patient education and shared decision-making [3, 4]. PsychMgmt sessions began preoperatively, with sessions carried out every two weeks. Following surgery, PsychMgmt sessions continued for 10-12 sessions, for up to approximately 3 months. Wellness Partners visited patient in-hospital for a "warm visit" whenever possible. MedOpt sessions were offered both preoperatively and postoperatively as well, to ensure that medication changes could be coordinated with both inpatient and outpatient teams prior to, and during, the inpatient stay. Sessions were conducted remotely via phone or secure web conference (Zoom), with optional in-person visits while the participant was hospitalized following surgery. PsychMgmt sessions lasted approximately 30-60 minutes, while MedOpt sessions lasted approximately 10-20 minutes.

Interventionist Training

Wellness Partners received training in psychological and medication-based treatments, including behavioral activation and medication optimization. Prior to beginning the study, they completed 4 weekly 1-hour training sessions, reviewing core behavioral activation strategies using previously published *Behavioral Activation Skills Assessment* modules. Wellness Partners also reviewed the PsychMgmt manual of procedures extensively prior to the start of the study.

The MedOpt pharmacy team received training led by two clinical pharmacy specialists across several weekly education sessions. Training included sessions on good clinical practices, use of the MedOpt manual of procedures, navigating the electronic health record (EHR) system, managing the intervention database, and communicating with compassion and empathy. Pharmacy students were also given supplemental readings on antidepressant dosing and medications that might be harmful to older adults.

Both PsychMgmt and MedOpt teams also attended weekly meetings to review progress, discuss challenges, and receive ongoing feedback. Across the study, both teams were supervised by a clinical psychologist, a PhD-level clinical social worker, and a geriatric psychiatrist.

Further details on PMHI components are available in Table 1.

Table 1. Details on PMHI Components.

**Intervention Bundle**	**Psychological Management**	**Medication Optimization**
Target	Patients	Clinicians and Patients
Interventionist	Wellness Partners	Pharmacists and pharmacy students
Description	A behavioral intervention that engages depressed and anxious patients in reinforcing activities or activities that are personally meaningful [1].	A pharmacologic intervention that deprescribes potentially inappropriate medications, adjusts suboptimal doses of antidepressants, and ensured continuation of antidepressants during transitions of care [5, 6].
Features	Cognitive-behavioral therapy (CBT)-based exercises where the Wellness Partner helps patients to generate a list of pleasant, reinforcing activities, and co-creates action plans.<br><br>Patient-centered treatment, based on patient preferences for modality (i.e. activities, scheduling).	Review of current medications for those that are eligible for deprescribing (e.g., strong centrally-acting anticholinergic and antihistaminergic drugs, benzodiazepines).<br><br>Identification of each patient’s likely need for medication adjustments, communication with outpatient providers about possible medication adjustments, and ongoing monitoring of patient responses to medication changes [7-9].
Rationale for including	Comparative efficacy and non-inferiority trials indicate that behavioral activation shows similar effectiveness to comprehensive CBT and can be delivered by less-trained staff (e.g., community health workers).	Medication optimization is strongly prioritized for the treatment of [11], as antidepressants are often prescribed at subtherapeutic doses and not adjusted [12], resulting in low effectiveness [13].<br><br>Strong centrally-acting anticholinergic and antihistaminergic drugs and

	Trials in medically ill patients show that behavioral activation complements medically indicated physical activation and exercise goals, with high feasibility and acceptability [10].	benzodiazepines can be harmful perioperatively, increasing falls and delirium [14-17].
Core active components	• Personalized rationale identification<br>• Values and goals assessment<br>• Activity scheduling<br>• Activity monitoring	• Review of medication list by wellness partner on 1st visit prior to surgery<br>• Determine the indication, duration of use, dose, and frequency of the medications of interest<br>• Evaluate each medication's eligibility for optimization or deprescription<br>• Discuss with medication optimization team<br>• Get buy-in from patient to contact initial prescriber<br>• Communicate recommendations to the patient<br>• Weekly review of any new medications<br>• Ensure that any medication optimization changes are reconciled during transitions of care and that the agreed-upon changes are implemented both pre-operatively and post-operatively
Modifiable components	• Activities: depending on patient needs and preferences<br>• Timing: Preoperative and postoperative<br>• Format: 1:1 session, in-person, over the telephone, or online<br>• Duration: 30-60 minutes<br>• Frequency: 1-4 sessions (presurgery); 10-12 sessions (postsurgery)<br>• Setting: Home and in-hospital	• Timing: Preoperative, in-hospital, and postoperative<br>• Format: 1:1 session, in-person, over the telephone, or online; telephone or online contact with outpatient providers<br>• Duration: 5 min<br>• Frequency: 1-4 sessions (presurgery); 10-12 sessions (postsurgery)<br>• Setting: Home and in-hospital

Control

Participants randomized to the control arm of each RCT received enhanced usual care (EUC). Along with standard perioperative care, participants within the EUC group were given evidence-based resources to mitigate anxiety and depression symptoms. These resources included information on “mindfulness and recovery from surgery,” “self-help for disrupted sleep,” and “training your brain” (i.e., cognitive training), and were selected based on prior work with older surgical patients and clinicians [18-20]. These resources were delivered in the form of paper handouts or emails. Study team members who delivered the resources informed participants that the resources were self-directed, or optional for participants to read or pursue. EUC participants had no interaction with Wellness Partners or MedOpt pharmacists.

1. Puspitasari, AJ, JW Kanter, AM Busch, et al., *A randomized controlled trial of an online, modular, active learning training program for behavioral activation for depression.* Journal of consulting and clinical psychology, 2017. **85**(8): p. 814.
2. Sinclair, S, TF Hack, CC MacInnis, et al., *Development and validation of a patient-reported measure of compassion in healthcare: the Sinclair Compassion Questionnaire (SCQ).* BMJ open, 2021. **11**(6): p. e045988.
3. Baqir, W, J Hughes, T Jones, et al., *Impact of medication review, within a shared decision-making framework, on deprescribing in people living in care homes.* Eur J Hosp Pharm, 2017. **24**(1): p. 30-33.
4. Jansen, J, V Naganathan, SM Carter, et al., *Too much medicine in older people? Deprescribing through shared decision making.* Bmj, 2016. **353**: p. i2893.
5. Lenze, EJ, E Lenard, M Bland, et al., *Effect of enhanced medical rehabilitation on functional recovery in older adults receiving skilled nursing care after acute rehabilitation: a randomized clinical trial.* JAMA network open, 2019. **2**(7): p. e198199-e198199.
6. Oughli, HA, H Lavretsky, J Karp, et al., *Optimizing Outcomes of Treatment-Resistant Depression in Older Adults (OPTIMUM): Study Design and Sample.* The American Journal of Geriatric Psychiatry, 2021. **29**(4): p. S33-S34.
7. Trivedi, MH and EJ Daly, *Measurement-based care for refractory depression: a clinical decision support model for clinical research and practice.* Drug Alcohol Depend, 2007. **88 Suppl 2**: p. S61-71.
8. Rush, AJ and ME Thase, *Improving Depression Outcome by Patient-Centered Medical Management.* Am J Psychiatry, 2018. **175**(12): p. 1187-1198.
9. Kok, RM and CF Reynolds, 3rd, *Management of Depression in Older Adults: A Review.* JAMA, 2017. **317**(20): p. 2114-2122.
10. Cowan, MJ, KE Freedland, MM Burg, et al., *Predictors of treatment response for depression and inadequate social support--the ENRICHD randomized clinical trial.* Psychotherapy and psychosomatics, 2008. **77**(1): p. 27-37.
11. Bao, Y, EP Post, TR Ten, et al., *Achieving effective antidepressant pharmacotherapy in primary care: the role of depression care management in treating late-life depression.* Journal of the American Geriatrics Society, 2009. **57**(5): p. 895-900.
12. Wang, PS, P Berglund, and RC Kessler, *Recent care of common mental disorders in the United States : prevalence and conformance with evidence-based recommendations.* J Gen Intern Med, 2000. **15**(5): p. 284-292.
13. Wang, PS, S Schneeweiss, MA Brookhart, et al., *Suboptimal antidepressant use in the elderly.* J Clin Psychopharmacol, 2005. **25**(2): p. 118-126.
14. Clegg, A and JB Young, *Which medications to avoid in people at risk of delirium: a systematic review.* Age Ageing, 2011. **40**(1): p. 23-29.
15. Ensrud, KE, TL Blackwell, CM Mangione, et al., *Central nervous system-active medications and risk for falls in older women.* J Am Geriatr Soc, 2002. **50**(10): p. 1629-1637.
16. Foy, A, D O'Connell, D Henry, et al., *Benzodiazepine use as a cause of cognitive impairment in elderly hospital inpatients.* J Gerontol A Biol Sci Med Sci, 1995. **50**(2): p. M99-106.

17. Lenze, EJ, A Iaboni, and JL Wetherell *Benzodiazepines in older adults: definite harms, doubtful benefits. (In response to: Benzodiazepine use and risk of Alzheimer's disease: case-control study. Billioti de Gage, et al.)*. The BMJ Online, 2014.
18. Lenze, EJ, S Hickman, T Hershey, et al., *Mindfulness-based stress reduction for older adults with worry symptoms and co-occurring cognitive dysfunction.* International journal of geriatric psychiatry, 2014. **29**(10): p. 991-1000.
19. Motter, JN, MA Pimontel, D Rindskopf, et al., *Computerized cognitive training and functional recovery in major depressive disorder: a meta-analysis.* Journal of affective disorders, 2016. **189**: p. 184-191.
20. Pereira, L, M Figueiredo-Braga, and IP Carvalho, *Preoperative anxiety in ambulatory surgery: The impact of an empathic patient-centered approach on psychological and clinical outcomes.* Patient education and counseling, 2016. **99**(5): p. 733-738.
