# Supplementary material for: Integrating Mental Health Into Surgical Care: A Qualitative Study of a Perioperative Mental Health Intervention
Source: Ann Surg Open. 2026 May 15;7(2):e675. doi: 10.1097/AS9.0000000000000675 (PMC13290208; doi:10.1097/AS9.0000000000000675)
Supplement: Supplementary file 2 [file as9-7-e675-s002.pdf]

Supplement 2. Participant Eligibility Criteria.

Participant Inclusion Criteria

Participants must meet the following criteria:

1. 60 years of age or older at the time of scheduled surgery;
2. Scheduled for a cardiac, oncologic, or orthopedic surgery at one of three eligible hospitals within our Midwestern healthcare organization
3. Presented with a score of 10 or greater on the Patient Health Questionnaire – Anxiety Depression Scale (PHQ-ADS).

Participant Exclusion Criteria

Participants may not participate in the study if they meet any of the following criteria:

1. Unable to read, speak, or understand English;
2. Presented with severe cognitive impairment, as demonstrated with a score of 10 or greater on the Short Blessed Test (SBT);
3. Determined to be ineligible due to conflicting study participation, as assessed by study principal investigator or patient's surgical team;
4. Determined to be ineligible due to previous participation within this study or the intervention feasibility study;
5. Presented with acute suicidal features, as assessed with a suicide safety screening, should the patient spontaneously report suicidal thoughts or score above a 0 on PHQ-9 item 9
