# Supplementary material for: Integrating Mental Health Into Surgical Care: A Qualitative Study of a Perioperative Mental Health Intervention
Source: Ann Surg Open. 2026 May 15;7(2):e675. doi: 10.1097/AS9.0000000000000675 (PMC13290208; doi:10.1097/AS9.0000000000000675)
Supplement: Supplementary file 3 [file as9-7-e675-s003.pdf]

Supplement 3. Semi-structured Interview Guide.

MEDICATION OPTIMIZATION AND CHANGES

Just to recap, [MedOpt pharmacy team member] reviewed your home medications with you and identified any targeted medications that have effects on the brain and could impact your mental health. The intention of this was to ensure dosages of certain medications are helpful to you and to limit medications that no longer benefit you or that could have undesirable effects on your brain.

1. Before beginning the study, did you know what medications you were taking and for what?
2. Do you frequently review your medications with your physician (PCP or geriatrician or psychiatrist)? How often do you have these medications reviews?
3. How was your experience with the medication optimization process of all your medications by the pharmacy team?
    a. What did you find helpful about the medication optimization?
4. Did you discuss any medication changes with [pharmacy team member] based on the review (all meds)?
5. How did this medication optimization/discussion differ from what you have done in the past?

NOTE: Go past this if MedOpt was implemented for the patient

6. Did [pharmacy team member] discuss any medication changes of [targeted medications] with you? If so, what changes were discussed?
7. How did you and [pharmacy team member] go about making changes to your medications?
    a. Did [pharmacy team member] explain why any changes should be made? Can you describe the medications that were changed, if any?
    b. Were changes made? Did those changes improve your stress and support your emotional well-being?
    c. What specific medications or dosages were changed? How were they changed?
    d. Were there any disagreements about which medications to change? What was the result of those disagreements?
    e. Did you experience any side effects due to any medication changes?
    f. What were the side effects? Were they communicated with [MedOpt pharmacy team member] and changed to help get rid of the side effects?
    g. Did you find changing your medications helpful? If so explain?
    h. Did you or [MedOpt pharmacy team member] share your newly updated medication list with your provider? If so, any feedback from the communication? Were they okay with the changes? If not, did you ask not to share and why?
    i. Did you stick to the changes that [MedOpt pharmacy team member] suggested?
        i. If so, how?
        ii. If not, what problems did you experience?

8. How effective was the review and discussion of the targeted medications [medications from their record] with [MedOpt pharmacy team member]? What did you find helpful about the medication review?
9. Did you experience any other problems with the medication optimization and changes made?
    a. If so, can you describe them further?
    b. Also, how could we address these in the future?
10. Do you have any other comments or suggestions regarding your medication optimization and discussion sessions?

PSYCHOLOGICAL MANAGEMENT AND CHANGES

To recap, our work with psychological management, behavioral activation, and changes involved assessing your emotional and behavioral responses to surgery and identify activities and goals to support their mental health during a difficult time. You worked on activities such as including setting and assessing values and goals, identifying activities to meet your goals, working towards those goals by scheduling these with [Wellness Partner] and monitoring these.

1. How was your experience with behavioral activation?
    a. How did you feel about each of these aspects of behavioral activation?
        i. Personal story and goals for recovery
        ii. Value and priority assessment
        iii. Activity scheduling
        iv. Activity monitoring and tracking
2. Was planning and acting upon behavioral changes helpful to you, and why?
    a. How did talking about your personal story and goals for the study help you cope with the recovery process and your mood?
    b. How did setting and assessing your values and priorities help you cope with the recovery process and your mood?
    c. How did identifying activities to meet your goals help you cope with the recovery process and your mood?
    d. How did working towards those goals by scheduling these with [Wellness Partner] help you cope with the recovery process and your mood?
3. Did [Wellness Partner] take into account "what mattered most" to you (e.g., outcome/goals/care preferences with regards to quality of life/functioning, enjoying life, connecting)?
    a. How did this affect your experience?
4. From your perspective, how effective were the behavioral change steps and activities in improving your well-being?
    a. How effective was behavioral activation in easing your stress/anxiety/depression surrounding surgery?
    b. Where did the behavioral changes help the most: pre-, post-operative periods?
    c. What did you like most about the behavioral changes?
    d. How did you discuss the activities and goals that you selected with [Wellness Partner]?

5. Do you feel that the timing and frequency of sessions were appropriate?
    a. Did you have sessions before or/and after surgery? (note: if not, please ask would it have helped before surgery?)
    b. Did the sessions before surgery help you prepare for the surgery and ease your anxiety and stress around surgery? Please explain how or why not.
    c. Did the sessions after surgery help your mental and physical recovery and well-being? Please explain how or why not.
    d. Would you change the number of sessions or how often you had them (frequency)?
        i. If so, how would you change them?
6. What are your thoughts on the format of the sessions?
    a. Do you prefer individual sessions or group sessions, and why?
    b. Was telephone effective or do you prefer in-person sessions? Would zoom option be something you would have been comfortable with?
        i. Why or not why?
7. Would you be willing to do these behavioral change activities if you were to need surgery again in the future?
    a. If so, why?
    b. If not, why not?
8. What were the advantages of these behavioral activities and changes based on it?
    a. What aspects of the behavioral change component would make you recommend these practices to friends/family?
9. What were the disadvantages of behavioral activation and changes?
    a. What made behavioral changes difficult to follow or adhere to?
    b. What did you like during these sessions? Please elaborate upon it.
    c. What did you dislike during these sessions? Please elaborate.
    d. What were your concerns and how can we improve upon addressing your concerns?
    e. Did you feel overwhelmed with these behavioral change activities at any point?
        i. Why or why not?

GENERAL QUESTIONS

1. Were you able to continue and maintain PMH intervention components on your own (after the intervention period)?
    a. Throughout the program, did you feel like continuing the sessions and the activities was viable and doable?
        i. Why or why not?
    b. Did anything or anyone help you to continue with the PMH intervention? Please elaborate.
    c. Did anything or anyone make it harder for you to continue with the PMH intervention? Please elaborate.
    d. Any suggestions to sustain the PMH intervention?
2. How confident do you feel that older adults having surgery at our hospital will accept and follow the PMH intervention, and why?
3. How could we “sell” the idea of this PMH intervention to others, especially to people who may be nervous or hesitant to do it?
4. How well do you feel that the PMH intervention could meet the needs of other adults that are going through similar stressors and surgeries? In what ways could this program improve their medication management and mood management throughout their surgical recovery?
5. Is there anything else missing from current practices that you think we could add to our PMH intervention?
6. Do you feel like we could assess progress with the program in any other way?
    a. If so, how?
7. What other educational materials would you want to see in our PMH intervention?
8. What was your experience with the research procedures – at the time of screening/consent?
    a. Did you encounter any issues with the assessments when you started the study, 1-month, 2-month and 3-month follow-ups?
        i. If so, what issues?
    b. Was there anything that you think did not work for you? Please elaborate.
9. Did you ever think about withdrawing or not participating anymore in this study?
    a. If so, why? What were the reasons?
    b. If not, what made you continue to participate?
10. What types of changes may we need to make for the intervention to be long-lived and available to all?
    a. Probe: What aspect of medication optimization and psychological management would make it difficult for you or someone else to adhere to it?
    b. Probe: How can we make sure that everyone independent of their socioeconomic status, race, ethnicity, gender, or other classification, is able to receive this intervention?

That’s everything! Do you have any questions for me?

For your payment, we will be loading your card with the $25 amount for your participation today. Thank you for your time!
