# Supplementary material for: Integrating Mental Health Into Surgical Care: A Qualitative Study of a Perioperative Mental Health Intervention
Source: Ann Surg Open. 2026 May 15;7(2):e675. doi: 10.1097/AS9.0000000000000675 (PMC13290208; doi:10.1097/AS9.0000000000000675)
Supplement: Supplementary file 4 [file as9-7-e675-s004.pdf]

Supplement 4. Periodic Reflection Focus Group Guide.

WELLNESS PARTNERS

General

1. How do patients react to your explanations about the principles and goals of behavioral activation when introducing this approach?
2. Can you describe your experience in using behavioral activation?
3. Success stories?
    a. Can you provide examples of how behavioral activation has improved their overall well-being of older surgical patients you have worked with?
4. What specific challenges do older surgical patients often face when it comes to engaging in meaningful activities?
5. How do you address these challenges in the context of behavioral activation?

Personalized Rationale

1. What is your experience with patients when identifying a personalized rationale?
2. What are some challenges with identifying and collaborating with the patient on identifying a personalized rationale?
3. How do you address potential feelings of isolation or loneliness that older surgical patients might experience, and how does behavioral activation help in mitigating these feelings?
4. What are some strategies that you use to address these?

Values and Goal Assessment

1. What are your experiences with getting patients to identify goals and values?
2. How do you assess their current behaviors, values and goals?
    a. Is this an easy step/ hard step?
3. How do you collaborate with them to set specific behavioral activation goals that are tailored to their individual needs and circumstances?
4. What techniques do you use to help older surgical patients set realistic goals and expectations for their behavioral activation activities, considering their current physical and emotional state?
5. Any challenges
6. How do you help patients maintain motivation and continue to engage in behavioral activation activities over time?
7. Can you explain your approach to addressing potential relapses or setbacks that clients may experience during behavioral activation?

Activity Scheduling

1. What are your experiences with scheduling activities with patients?
2. How do you address potential reluctance or resistance from older patients who may be apprehensive about trying new activities or engaging in social interactions?
3. Typically how many activities do you try to schedule during each session?
4. What strategies do you use to motivate and encourage older surgical patients to engage in activities despite potential pain, fatigue, or discomfort?
5. Do you use forms to support scheduling ? What materials do you use?
6. What strategies do you employ to help patients identify and overcome barriers to engaging in meaningful and rewarding activities?
7. Challenges?

Activity Tracking

1. What are your experiences with patients tracking or monitoring their activities and reporting back in the next session?
2. How do you use this information to guide your sessions?
3. Can you describe your approach to monitoring progress and adjusting behavioral activation plans as needed for older surgical patients during their recovery process?
4. Do they document their activities and are they reviewed?
5. Do you see any benefits in tracking activities?
6. What are some challenges that you face when there is no documentation?

Adaptations

1. How do you adapt behavioral activation to address the unique challenges and needs of patients with different mental health conditions, such as depression, anxiety, or bipolar disorder?
2. How do you adapt the intervention components of BA based on:
    a. Patient individual differences?
    b. Pre- vs. post-op differences in each of the components
    c. Active vs. less-active patients (physical and cognitive limitations)?
    d. Employed vs. retired patients?

    e. Cultural differences
    f. Surgical context
3. Have you experienced any differences in how BA is delivered between the surgical cohorts?
4. Any changes made to implementation plan?
    a. Timing
    b. Session frequency?
    c. Modality?
5. How have these adaptations helped?
6. What are the challenges you face when you change the implementation plan?

Interventionist Process

1. Can you describe your process to prepare for these sessions?
2. What techniques do you use to track and measure your patients' progress when using behavioral activation?
3. How do you help patients maintain motivation and continue to engage in behavioral activation activities over time?
4. Can you explain your approach to addressing potential relapses or setbacks that clients may experience during behavioral activation?
5. What resources or tools do you use to support patients in implementing behavioral activation in their daily lives?
6. Any training needed? Gaps in training you experience and wished for more training?

Demonstrating Compassionate Care

1. Can you describe your approach to providing compassionate care to patients?
2. How do you demonstrate empathy and understanding when working with patients who may be facing challenging or emotional issues?
3. What strategies do you use to create a safe and non-judgmental environment that promotes trust and open communication with patients?
4. How do you involve patients in the decision-making process regarding their treatment and care, taking into account their values and preferences?
5. Can you provide examples of times when your compassion and empathy made a significant difference in a patient's experience or outcomes?

PHARMACY TEAM

Medication Optimization

1. How do you explain medication optimization to CPMH patients? Are there other terms that may be more appealing to patients?
2. What are your experiences with MO process?
    a. Success stories/ positive experiences?
    b. Can you provide examples of how you've successfully optimized psych medications for older patients, including any improvements in their quality of life or mental health outcomes?
    c. How do you collaborate with other healthcare providers, such as primary care physicians and specialists, to ensure comprehensive and coordinated care for older patients taking psych medications? How is your experience with that?
3. Unsuccessful stories?
    a. Any challenges with the initial session and follow-up sessions?
4. Are there specific types of medications that are more challenging to patients to implement your changes?
    a. What do you do in those cases? Do you offer alternatives?
5. Have there been patient cases where patients were not happy with the change of medications proposed by you?
    a. Examples, and why?
    b. What do you do in these cases?
6. Challenges with other healthcare providers collaboration and coordination of medications? How is this addressed?

Workflow

1. How do you prepare for these sessions? May be different or same for you all.
    a. What works well for you?
    b. Challenges?
2. What strategies do you use to communicate potential side effects or adverse reactions to psych medications in older patients, considering their vulnerability to such issues?

   a. Can you provide information on how you educate older patients and their caregivers about the proper use, potential side effects, and other important considerations of psych medications?
   b. How do you address and discuss potential challenges of polypharmacy (multiple medications) that are common among older adults, especially when psych medications are involved?
3. Can you describe your approach to ensure that you have followed up with patients while on updated CPMH optimized list of medications between sessions?
4. What techniques do you use to track and measure your patients' reactions and response to the med changes made?
   a. Would there be differences based on the type of medications (harmful vs. antidepressant)?
5. Do you wish for or need any additional training or review of anything to be comfortable with older surgical patients?

Adaptations

1. Any changes made to implementation plan?
   a. Timing (before surgery and after surgery) – works?
   b. Session freq? Avg number?
   c. Modality?
2. How have these adaptations helped?
3. What are the challenges you face when you change the implementation plan?

Demonstrating Compassionate Care

1. Can you describe your approach to providing compassionate care during these MO sessions to patients?
2. How do you demonstrate empathy and understanding when working with patients who may be facing challenging or emotional issues?
3. How do you involve patients in the decision-making process regarding their medication changes?
4. Can you provide examples of times when your compassion and empathy made a significant difference in a patient's experience or outcomes?
