# Supplementary material for: Integrating Mental Health Into Surgical Care: A Qualitative Study of a Perioperative Mental Health Intervention
Source: Ann Surg Open. 2026 May 15;7(2):e675. doi: 10.1097/AS9.0000000000000675 (PMC13290208; doi:10.1097/AS9.0000000000000675)
Supplement: Supplementary file 5 [file as9-7-e675-s005.pdf]

Supplement 5. Conceptual Model Themes and Subthemes.

**Themes and Subthemes**	**Definition**
Perceived value of PMH intervention	The perceived effectiveness of psychological management (PsychMgmt) and medication optimization (MedOpt) within the PMH intervention on patients' mental health and recovery process
Motivation	The extent and ways in which the PsychMgmt component provided a sense of accountability, self-awareness, and motivation in patients during recovery through goal setting and activity scheduling
Cognitive restructuring	The extent to which the PsychMgmt component introduced patients to new activity ideas and reframed their mindset to set realistic expectations for recovery
Safe practice	The extent to which MedOpt helped patients stop or change medications due to side effects or drug interactions
Education	The degree of thoroughness of MedOpt that allowed patients to better understand their medications
**Previous patient experiences**	The degree to which patients' previous healthcare experiences affected their beliefs about the PMH intervention
Prior surgery and recovery	The extent to which patients valued their surgery and recovery experiences with the PsychMgmt component by comparing with previous surgery and recovery experiences
Prior medication reviews	The extent to which patients valued their MedOpt medication reviews by comparing them to previous medication reviews with their outpatient providers
Prior relationship with care team	The extent to which patient relationships with outpatient care teams or prior surgical care teams affected their perception of the PMH intervention and interventionists
Pre-operative quality of life	The comparison between a patient's pre-operative physical and mental baseline and their post-operative status
**Patient experiences with interventionist team**	The patient beliefs about the clinical teams involved in the PMH intervention (wellness partner and pharmacy team) and the extent to which they collaborated with patients' outpatient providers and surgical teams
Personalized support	The degree to which the wellness partner and pharmacy team provided a safe space for patients to discuss their needs and personalized care to the patients' goals

Compassionate, collaborative care	The degree to which the wellness partner and pharmacy team showed empathy and kindness to patients
Interdisciplinary coordination	The level of coordination among the wellness partner, pharmacy team, outpatient providers, and surgical team when introducing the PMH intervention, decision-making on the patients' behalf, and setting follow-ups
Rapport building	The process of establishing a mutual and trusting relationship between patient and interventionist through personal connection and communication
Nature of surgery and recovery	The unique characteristics of each type of surgical procedure that impacted patients' mental and physical preparation and recovery
**Implementation characteristics of PMH intervention delivery**	The structures of the PMH intervention such as the delivery method, timing, frequency, feasibility, and willingness to repeat
Adherence to intervention	The factors influencing whether patients considered withdrawing from the PMH intervention and the reasons they chose to continue
Patient-centered delivery	The preferred format of the sessions, including individual vs. group, pre-operatively vs. post-operatively, timing/frequency, and preferred delivery method (telephone, Zoom, or in-person)
Sustainability	The extent to which patients believed that the PMHI was acceptable, appropriate, and feasible long-term, and whether they felt that they could continue to maintain the PMHI after the end of the program/whether they would be willing to repeat the PMHI for future procedure
